# Excessive Dietary Lipid Affecting Growth Performance, Feed Utilization, Lipid Deposition, and Hepatopancreas Lipometabolism of Large-Sized Common Carp (*Cyprinus carpio*)

**DOI:** 10.3389/fnut.2021.694426

**Published:** 2021-07-13

**Authors:** Ze Fan, Jinnan Li, Yuanyuan Zhang, Di Wu, Xianhu Zheng, Chang'an Wang, Liansheng Wang

**Affiliations:** Key Laboratory of Aquatic Animal Diseases and Immune Technology of Heilongjiang Province, Heilongjiang River Fisheries Research Institute, Chinese Academy of Fishery Sciences, Harbin, China

**Keywords:** common carp, lipid requirement, growth performance, lipid deposition, hepatopancreas lipometabolism

## Abstract

An 82-day study was conducted to assess the effect of the dietary lipid levels on growth performance, feed utilization, lipid deposition, and hepatopancreas lipometabolism of large-sized common carp (*Cyprinus carpio*). Six isonitrogenous (300 g/kg protein) pelletized diets with different dietary lipid levels (30, 60, 90, 120, 150, and 180 g/kg) were fed in triplicate to fish groups with 75 individuals (with an initial mean weight of 247.00 ± 16.67 g). The results showed that there was a significant increase in weight gain (WG) rate (WGR), specific growth rate (SGR), and protein efficiency ratio (PER) as dietary lipid levels increased from 30 to 60 g/kg (*p* < 0.05) and then there was a decline. Feed conversion rate (FCR) was observed to be significantly lower in 60 g/kg lipid treatments (*p* < 0.05). Muscle crude protein contents were obtained to be significantly higher in 60 and 90 g/kg treatments (*p* < 0.05). The crude lipid content in the hepatopancreas increased significantly with an increase in dietary lipid levels (*p* < 0.05). The expression of lipoprotein lipase (LPL) and carnitine palmitoyltransferase-1 (CPT1) in the hepatopancreas was significantly downregulated with an increase in dietary lipid levels while the expression of growth hormone (GH), insulin-like growth factor-1 (IGF-1), fatty acid synthase (FAS), acetyl-CoA carboxylase-1 (ACC-1), and sterol regulatory element binding protein (SREBP) was upregulated first in 30 and 60 g/kg lipid treatments and then downregulated significantly in other treatments. The results revealed that excess dietary lipid supplements (more than 60 g/kg) would inhibit WG and would aggravate the lipid decomposition in the hepatopancreas. Based on WGR and FCR, the dietary lipid levels of 59.5 and 70.4 g/kg were optimal for growth performance and feed utilization of large-sized common carp.

## Introduction

For decades, the exploration of an approach to minimizing the dietary protein consumption and simultaneously maximizing the protein utilization efficiency has been extremely imperative for the sustainable development of the aquaculture feed industry (1–3). Investigators have begun to perform feasibility studies on the protein-sparing effect of lipids in fish diets (4–7). In addition to the prominent protein-sparing effect, the traditional functions of lipids involve the provision of high energy values, promotion of the absorption of fat-soluble vitamins, inhibition of dust, improvement of the palatability, etc., which are important for the feed industry (8). Moreover, from a fish nutrition perspective, dietary lipids play a crucial role in furnishing essential fatty acids, phospholipids, sterols, and fat-soluble vitamins and maintaining the stability of biological structures and cell membrane functions (9–11). If a shortage occurs in dietary lipid supplements, then metabolic disturbance may be observed in fish along with a decrease in the efficiency of feed protein and the lack of fat-soluble vitamins and essential fatty acids (12). Thus, there has been a visible tendency to supply higher concentration of lipids in commercial diets.

However, high concentrations of lipids have been included in commercial diets, which have created controversy among some researchers and fish farmers (13). Excess dietary lipid supplements could bring about a diminution in feed consumption or feed ingestion rate by fish, thereby limiting the growth performance of the farmed fish (14). Moreover, there are misgivings on the fact that an excessive amount of dietary lipid could increase fat deposition in the liver and muscle, thereby affecting the flavor and nutritional qualities of the product (15, 16). Meanwhile, for many other fish species, an excess of dietary lipids could contribute to oxidative stress, accompanied by negatively affecting survival, growth, disease resistance, stress response, and lipid metabolism (17). In addition, for fish, the dietary lipid requirement varies distinctly with different species, body weights, life stages, and environmental conditions (18). Under such circumstances, appropriate dietary lipid supplements for different fish species should be confirmed to ensure optimal growth and production quality and to prevent dietary protein from being used as energy.

Common carp (*Cyprinus carpio*), with the strong adaptive capability to both environment and food (19, 20), is commercially important (21) and represents the third most widely cultivated freshwater fish species in the globe (22). The common carp production in 2018 was 2,962,218 tons in China, which ranked at the fourth position out of a total of 25 species evaluated (23). However, to our knowledge, the available information on the dietary lipid requirement for common carp was limited to juvenile common carp with the body weights below 20 g and on growing common carp with the weights ranging from 20 to 100 g (24). Xu et al. (24) obtained the results from the study on common carp [an initial body weight (IBW) of 5.51 ± 0.05 g] in which the optimum dietary lipid requirement of juvenile common carp was 8% at 16°C and 11% at 23°C in terms of immunity. When the weight of common carp reached 15 g, a diet of them containing 80 g/kg lipids was optimal for growth performance and health status (25). Additionally, juvenile common carp with an IBW of 105 g showed the best growth performance with the dietary lipid level of 44 g/kg (26). However, knowledge of the dietary lipid requirements of common carp with an IBW of more than 200 g is limited, and the detailed studies on this topic are currently lacking. Regarding the current situation, with an aim to optimize the dietary lipid requirement of large-sized common carp of more than 200 g and further strengthen the precise nutrition regulation in the breeding process of common carp, the effects of practical diets with different lipid contents on growth performance, body composition, and hepatopancreas lipometabolism of large-sized common carp were evaluated.

## Materials and Methods

### Preparation of Feedstuff and Chemical Reagent

Fish meal (crude protein, 670 g/kg diet and crude lipid, 100 g/kg diet), soybean meal (crude protein, 440 g/kg diet and crude lipid, 15 g/kg diet), and wheat middlings (crude protein, 130 g/kg diet and crude lipid, 12 g/kg diet) were purchased from Hehe Feed Co., Ltd (Dongguan, China). Soybean oil was purchased from Jiusan Oils & Grains Industries Group Co., Ltd. (Heilongjiang, China).

Hydrochloric acid (analytical reagent, AR), sulfuric acid (AR), copper sulfate pentahydrate, potassium sulfate (AR), sodium hydroxide (AR), boric acid (AR), ammonium sulfate (AR), methyl red, bromocresol green, and anhydrous ethanol were obtained from Sinopharm Chemical Reagent Co., Ltd. (Shanghai, China). Petroleum ether (40–60°C) for the determination of crude lipid in the diets, whole body, dorsal white muscle, and hepatopancreas was obtained from Sinopharm Chemical Reagent Co., Ltd. (Shanghai, China).

### Feed Formulation and Preparation

Diet ingredients and compositions supplied to common carp are listed in Table 1. Six dietary treatments containing six lipid levels of 30, 60, 90, 120, 150, and 180 g/kg were formulated according to the National Research Council (NRC) (11) (Table 1). Fish meal and soybean meal were regarded as protein sources. Soybean oil was regarded as the lipid source. As the lipid level rose, the content of wheat middling decreased correspondingly to maintain the energy content. Carboxymethylcellulose sodium (CMC-Na) and cellulose were treated as a filler. The dry feed ingredients were ground with a grinder and sieved, weighed, and thoroughly mixed in a horizontal mixer (SLHY-1, Dashiqiao Bao's Feed Machinery Factory, Yingkou city, Liaoning Province, China). Soybean oil, CMC, and cellulose were appended and adequately blended to make dough, and vitamin-mineral premixes and trace mineral premix were appended. Moist diets were made into 3-mm-sized pellets by a pelletizer (GYJ-250B, Dashiqiao Bao's Feed Machinery Factory). The dry diets were stored at −20°C until their use.

**Table 1:** Ingredients and proximate composition of experimental diets (g/kg of dry matter basis).

**Ingredients^**a**^ (g/kg)**	**Experimental diets**					
	**30 g/kg**	**60 g/kg**	**90 g/kg**	**120 g/kg**	**150 g/kg**	**180 g/kg**
Soybean meal	550	550	550	550	550	550
Wheat middling	310	280	250	220	190	190
Fish meal	50	50	50	50	50	50
Soybean oil	15	45	75	105	135	165
Carboxymethylcellulose sodium (CMC-Na)	10	10	10	10	10	10
Choline chloride	5	5	5	5	5	5
Dicalcium phosphate	25	25	25	25	25	25
Vitamin premix^a^	3	3	3	3	3	3
Trace mineral premix^b^	2	2	2	2	2	2
Cellulose	150	120	90	60	30	0
**Proximate composition**						
Crude protein	300.2	298.9	300.3	299.7	299.1	300.1
Crude lipid	30.5	60.6	90.4	121.3	152.5	178.9
Crude ash	67.6	67.4	68.3	68.6	68.1	67.7
Carbohydrate (NFE)^c^	409.95	393.45	376.95	360.45	343.95	343.95
Estimated gross energy/(kcal/100 g)^d^	366.12	386.93	409.26	430.46	451.66	480.54

a *Vitamin mixture (g/kg mixture) supplied by Guangdong Hyint Biotechnology Group Co., Ltd. (Guangdong, China): vitamin A (VA) 8,000 IU, vitamin C (VC) 100 mg, vitamin D_3_ (VD_3_) 3,000 IU, vitamin E (VE) 60 mg, vitamin K_3_ (VK_3_) 5 mg, vitamin B_1_ (VB_1_) 15 mg, vitamin B_2_ (VB_2_) 30 mg, vitamin B_6_ (VB_6_) 15 mg, and vitamin B_12_ (VB_12_) 0.5 mg.*

b *Trace mineral mixture (mg/g mixture) supplied by Guangdong Hyint Biotechnology Group Co., Ltd. (Guangdong, China): nicotinamide 175 mg, d-biotin 2.5 mg, inositol 1,000 mg, folic acid 5 mg, pantothenic acid 50 mg, zinc (Zn) 60 mg, copper (Cu) 3 mg, iron (Fe) 25 mg, manganese (Mn) 15 mg, iodine (I) 0.6 mg, and magnesium (Mg) 0.7 mg.*

c *NFE, Nitrogen-free extract.*

d *Estimated gross energy/ (kcal/100 g) = (4 × crude protein %) + (9 × lipid %) + (4 × carbohydrate %)*.

### Fish Rearing

The feeding trial was carried out in the floating net cages (2 × 2 × 2 m) in Hulan Experimental Station of Heilongjiang Fisheries Research Institute (Harbin, China). Large-sized common carp were also obtained from the Hulan Experimental Station of Heilongjiang Fisheries Research Institute (Harbin, China), and were fed with a commercial diet (320 g/kg crude protein and 60 g/kg crude lipid) for 2 weeks before the feeding trial to acclimate to the experimental conditions. Prior to the beginning of the feeding trial, six triplicate groups of fish with healthy status and of uniform size (25 individuals per replicate with an initial mean weight of 247.00 ± 16.67 g, 1 year of age) were randomly assigned into the floating net cages, respectively. Throughout the 82-day feeding trial, the diets were provided to the fish at the rate of 3–4% of the body weight thrice at 8:00 a.m., 13:00 p.m., and 17:30 p.m. every day for 82 days. Meanwhile, the water temperature (28–32°C), total ammonia (0–0.20 mg/L), and dissolved oxygen (5.8–6.2 mg/L) were monitored daily. All water quality parameters were determined by using a multi-parameter water quality analyzer (HQ40D) (HACH Company, Loveland, CO, USA).

### Calculations of Growth Indexes and Sampling

All fishes in the cage were weighed and counted to assess the survival rate (SR), weight gain (WG), feed conversion rate (FCR), specific growth rate (SGR), protein efficiency ratio (PER), and condition factor (CF) after starving for 24 h, according to the following formulas:

SR (%) = 100 × final number (fish)/initial number (fish); Feed intake (FI, g/fish) = total feed intake per cage (g)/(number of fish^*^82 days); WGR (%) = 100 × [final weight (g)–initial weight (g)]/final weight (g); Feed conversion rate (FCR) = 100 × feed supplied (g)/WG (g); SGR (%/d) = 100 × [(ln final weight–ln initial weight)/82 days]; PER (%) = 100% × [final weight (g)–initial weight (g)]/[total feed intake (g) × content of dietary protein (%)]; CF (g/cm^3^) = weight (g)/length^3^ (cm); Three fishes per cage (nine fishes per diet treatment) were randomly allotted, anesthetized with MS-222 (0.1 g/L) (27), and weighed and measured, and the blood samples were drawn from the caudal vein. And then these fishes were dissected to acquire the hepatopancreas for assessing the hepatosomatic index (HSI), according to the following formulas: HSI (%) = 100 × [wet weight of hepatopancreas (g)/final weight (g)], and the hepatopancreas was stored at −20°C for chemical analysis and at −80°C for gene expression analysis, respectively. Simultaneously, the dorsal white muscles of these fishes were acquired for the composition analysis, which were stored at −20°C. Additionally, three fishes from each cage (nine fishes per diet treatment) were randomly chosen and ground for later proximate composition analysis of the whole body.

### Sample Preparation and Biochemical Analysis

Proximate composition analysis of the diets, whole body, and dorsal white muscle were carried out according to the Association of Official Analytical Chemists (AOAC) (2006) (28) for moisture (drying at 105°C for 24 h), crude protein (Kjeldahl method; N × 6.25), crude ash, and crude lipid (the Soxhlet method).

Serum samples were isolated by high-speed centrifugation at 2,500 rpm at 4°C for 20 min. All indices involved in the serum were assayed using specific analytical procedures and commercially available kits, which were supplied by Nanjing Jiancheng Bioengineering Institute, Nanjing, China. Among these, total cholesterol (TCHO) was determined by CHOD PAP-CDC method (Cat. No. A111-2-1). Triacylglycerol (TG) was measured by GPD-PAP method (Cat. No. A110-2-1). High-density lipoprotein-cholesterol (HDL-C) and low-density lipoprotein-cholesterol (LDL-C) were measured according to the selective inhibition method (Cat. Nos. A112-1-1 and A113-1-1).

### Determination of the Relative Gene Expressions

Referring to the manufacturer's instructions of RNAiso Plus (TaKaRa, Dalian, Liaoning, China), total RNA extraction of the hepatopancreas samples was carried out. The isolated RNA was spectrophotometrically quantified using a NanoDrop 2000 (Thermo Fisher Scientific, Waltham, MA, USA) and electrophoresed on a 1% denaturing agarose gel to test the integrity. About 1,000-ng RNA was reverse transcribed into complementary DNA (cDNA) following the manufacturer's instructions of TaKaRa PrimeScript^TM^ RT reagent Kit with genomic DNA (gDNA) Eraser (Perfect Real Time) (Code No.: RR047A) (Dalian Takara Company, Dalian City, Liaoning Province, China). After the cDNA templates diluted into 50 ng/μl by DEPC water, the targeted gene expression levels in the cDNA templates were measured by quantitative real-time PCR conducted on ABI 7500 real-time PCR machine (ABI, Applied Biosystems, Waltham, MA, USA) with the TaKaRa SYBR® Premix Ex Taq^TM^ (Tli RNaseH Plus) (Code No.: RR420A) (Dalian Takara Company, Shiga, Japan). The reaction was conducted in a 20-μl volume containing 10 μl SYBR® Premix DimerEraser (2 ×), 0.4 μl PCR Forward Primer, PCR Reverse Primer, 2 μl cDNA template (≈100 ng), 7 μl RNase free dH_2_O according to the manufacturer's protocol of the TaKaRa SYBR® Primix Ex TaqTM (Tli RNaseH Plus, Shiga, China) (Code No.: RR420A). The primers were designed based on the obtained sequences from the National Center for Biotechnology Information (NCBI) and listed in Table 2. β-actin was regarded as a reference gene to normalize cDNA loading. Based on the specific gene standard curves, the amplification efficiency of the target and the housekeeping gene was calculated, which was generated from 10 fold serial dilutions. The thermal cycle program of quantitative reverse transcriptase PCR (qRT-PCR) was as follows: 95°C for 30 s (pre-degeneration stage), 40 cycles at 95°C for 5 s, 60°C for 34 s (cycling stage), then 95°C for 15 s, 60°C for 1 min, 95°C for 30 s, and 60°C for 15 s (melt curve stage). The comparative CT method (2^−ΔΔCt^) method was used to calculate the relative expression levels of each gene.

**Table 2:** Primers used for the quantitative reverse transcriptase -PCR (qRT-PCR).

**Gene name**	**Primer sequence (5^′^-3^′^)**	**Product size (bp)**	**Accession number**
GH^1^	F:ATCTTCCCTCTGTCTTTCTGC; R:AAGTCGGCCAGCTTCTCA	144	M27000.1
IGF-1^2^	F: AGACAGCCCAAGGACAGCA; R: TACAGTGGAGCACATCTCTGGAA	204	HM565013.1
FAS^3^	F:GACAGGCCGCTATTGCTATT; R:TGCCGTAAGCTGAGGAAATC	110	GQ466045.1
LPL^4^	F:CGCTCCATTCACCTGTTCAT; R:GCTGAGACACATGCCCTTATT	105	FJ716101.1
CPT-1^5^	F:CAGATGGAAAGTGTTGCTAATGAC; R:TGTGTAGAAGTTGCTGTTGACCA	168	JQ361077.1
SREBP^6^	F:CGTCTGCTTCACTTCACTACTC; R:GGACCAGTCTTCATCCACAAA	141	XM_019073316.1
ACC^7^	F:TTCACTGGCGTATGAGGATATC; R:TCCACCTGTATGGTTCTTTGG	105	XM_019096370.1
β-actin	F:GGCAGGTCATCACCATCGG; R:TTGGCATACAGGTCTTTACGG	107	M24113.1

1 *Growth hormone (GH);*

2 *Insulin-like growth factor-1 (IGF-1);*

3 *Fatty acid synthase (FAS);*

4 *Lipoprotein lipase (LPL);*

5 *Carnitine palmitoyltransferase-1 (CPT1);*

6 *Sterol regulatory element binding protein (SREBP); and*

7 *Acetyl-CoA carboxylase-1 (ACC-1)*.

### Statistical Analysis

The results were presented as the mean ± SD. All data were subjected to a one-way ANOVA by Duncan's multiple range tests to evaluate significant differences among the treatments at *p* < 0.05 using SPSS statistics version 22.0 (SPSS Inc., Chicago, IL, USA). A second-order polynomial regression model was used to estimate the optimal dietary lipid level for large-sized common carp.

## Results

### Growth Performance and Feed Utilization

Survival rates were ranged from 98.67 to 100.00%. Significant enhancements in WGR and SGR were observed by increasing dietary crude lipid levels from 30 to 60 g/kg (*p* < 0.05), and further increase of the dietary lipid levels from 90 to 180 g/kg resulted in a significant decrease in growth performance (*p* < 0.05). The optimal growth performance of fish with the highest WGR and SGR was found in the 60 g/kg dietary lipid treatment, with a significant difference in comparison with other groups (*p* < 0.05). The highest PER, FI, and lowest FCR were observed in the 60 g/kg dietary lipid treatment, with a significant difference in comparison with 120, 150, and 180 g/kg dietary lipid treatments, respectively (*p* < 0.05). The highest HSI in the 180 g/kg crude lipid group was observed, and the highest CF was observed in the 150 g/kg crude lipid group, with significant differences in comparison to the 30 g/kg dietary lipid treatment (*p* < 0.05; Table 3).

**Table 3:** Growth performance and feed utilization of large-sized common carp fed the diets containing different lipid levels^*^.

**Items**	**Varying levels of dietary lipid**					
	**30 g/kg**	**60 g/kg**	**90 g/kg**	**120 g/kg**	**150 g/kg**	**180 g/kg**
IBW^1^	236.67 ± 3.52	242.67 ± 12.67	260.67 ± 12.02	241.33 ± 8.67	258.67 ± 10.97	242.00 ± 4.62
FBW^2^	587.69 ± 8.14^bc^	668.59 ± 34.67^a^	651.74 ± 26.39^ab^	583.92 ± 7.87^c^	610.17 ± 18.19^abc^	556.07 ± 8.69^c^
SR^3^	100.00 ± 0.00	98.67 ± 1.33	100.00 ± 0.00	98.67 ± 1.33	100.00 ± 0.00	98.67 ± 1.33
WGR^4^	148.33 ± 0.61^b^	175.53 ± 1.32^a^	150.17 ± 5.31^b^	142.37 ± 10.11^bc^	136.17 ± 6.79^cd^	129.82 ± 2.00^d^
PER^5^	1.70 ± 0.07^a^	1.74 ± 0.07^a^	1.67 ± 0.08^a^	1.54 ± 0.02^b^	1.52 ± 0.02^b^	1.38 ± 0.03^c^
SGR^6^	1.11 ± 0.00^b^	1.24 ± 0.01^a^	1.12 ± 0.03^b^	1.08 ± 0.05^bc^	1.05 ± 0.03^cd^	1.02 ± 0.01^d^
FI^7^	8.96 ± 0.23^b^	9.84 ± 0.42^a^	9.47 ± 0.25^ab^	9.38 ± 0.15^ab^	9.11 ± 0.08^ab^	8.92 ± 0.01^b^
FCR^8^	2.10 ± 0.05^bc^	1.91 ± 0.04^d^	1.99 ± 0.06^cd^	2.17 ± 0.05^b^	2.19 ± 0.02^b^	2.40 ± 0.03^a^
HSI^9^	1.67 ± 0.23^b^	1.73 ± 0.21^ab^	1.97 ± 0.21^ab^	1.89 ± 0.24^ab^	2.03 ± 0.27^ab^	2.44 ± 0.23^a^
CF^10^	3.06 ± 0.12^b^	3.18 ± 0.11^ab^	3.11 ± 0.13^ab^	3.09 ± 0.13^b^	3.59 ± 0.28^a^	3.43 ± 0.10^ab^

^*^Values are the mean ± SD (n = 3) of three replicates.

*Values within the same row with different small letters in superscript are significantly different (p < 0.05).*

*^1^Initial mean body weight (IBW, g per fish); ^2^Final mean body weight (FBW, g per fish); ^3^Survival rate (SR, %); ^4^Weight gain rate (WGR, %); ^5^Protein efficiency ratio (PER, %); ^6^Specific growth rate (SGR, %/d); ^7^Feed intake (FI, g/fish); ^8^Feed conversion rate (FCR); ^9^Hepatosomatic index (HSI, %); and ^10^Condition factor (CF, g/cm^3^)*.

**Figure 1:**
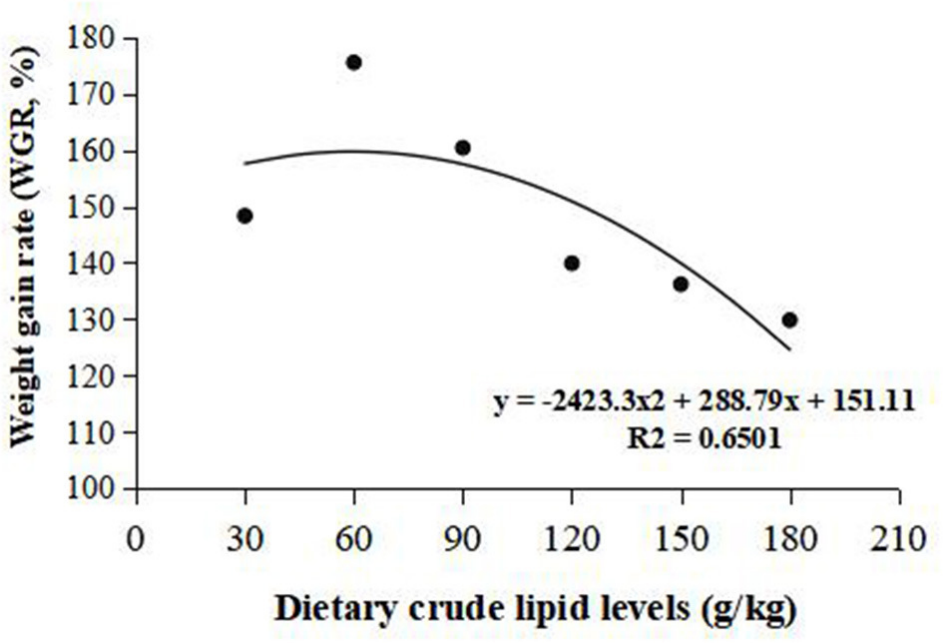
Dietary crude lipid requirement for large-sized common carp based on the second-order polynomial regression analysis of weight gain rate (WGR) against dietary lipid levels (g/kg). WGR (%).

**Figure 2:**
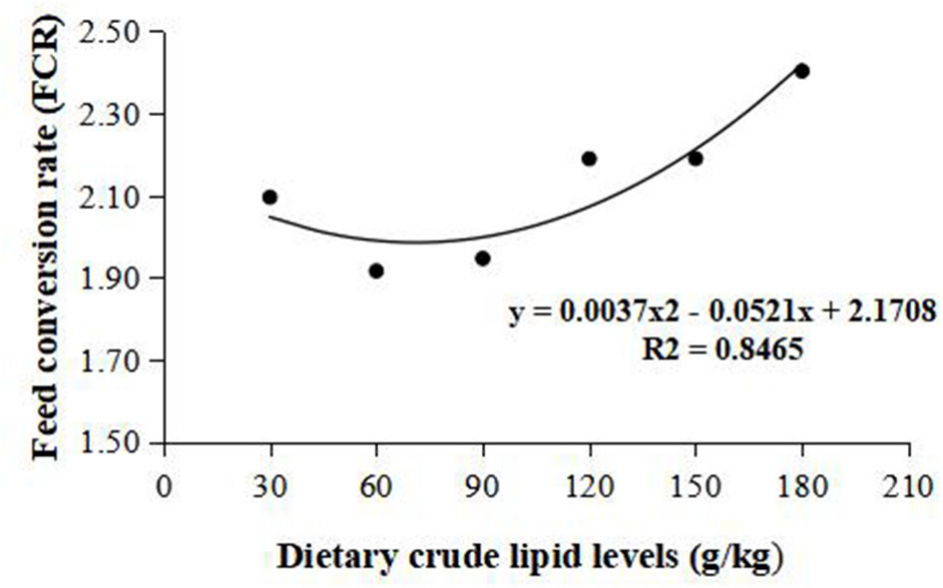
Dietary crude lipid requirement for large-sized common carp based on the second-order polynomial regression analysis of feed conversion rate (FCR) against dietary lipid levels (g/kg). FCR, Feed conversion rate.

Weight gain rate and FCR were used in the second-order polynomial regression analysis for the estimation of optimum dietary lipid requirement, and the analysis showed that the optimal dietary lipid requirement for maximum growth of large-sized common carp was, respectively, estimated to be 59.5 and 70.4 g/kg of dry matter (Figures 1, 2).

### Whole-Body, Dorsal White Muscle, and Hepatopancreas Compositions

The highest whole-body crude protein content was obtained in fish fed the 60 g/kg dietary lipid treatment. The highest whole-body crude lipid content was observed in fish fed the 180 g/kg dietary lipid treatment. The highest muscle crude protein content was obtained in fish fed the 90 g/kg dietary lipid treatment, only with no significant difference in comparison to the 60 g/kg dietary lipid treatment (*p* < 0.05). The muscle crude lipid content in the 180 g/kg dietary lipid treatment was significantly higher than that in other dietary lipid treatments (*p* < 0.05). For the hepatopancreas compositions, the crude lipid content increased with an increase in dietary protein (*p* < *0.05*), and the highest crude lipid content of the hepatopancreas was observed in fish fed the 180 g/kg dietary lipid treatment, with significant differences from the other dietary lipid treatments (*p* < 0.05). No significant difference was found in the content of moisture of the hepatopancreas in all treatment groups (*p* > 0.05; Table 4).

**Table 4:** Whole-body, dorsal white muscle, and hepatopancreas compositions of large-sized common carp fed the diets containing different lipid levels^*^.

**Items**	**Varying levels of dietary lipid**					
	**30 g/kg**	**60 g/kg**	**90 g/kg**	**120 g/kg**	**150 g/kg**	**180 g/kg**
**Whole-body**						
Crude protein/%	14.84 ± 0.21	15.37 ± 0.33	15.29 ± 0.74	14.59 ± 0.18	14.41 ± 0.15	14.20 ± 0.54
Crude lipid/%	6.97 ± 0.49	6.06 ± 0.34	6.70 ± 0.15	6.58 ± 0.35	7.30 ± 0.64	7.73 ± 0.25
Moisture/%	72.37 ± 1.13	73.81 ± 1.88	73.34 ± 0.41	74.53 ± 1.74	74.35 ± 1.84	73.13 ± 1.25
Ash/%	2.87 ± 0.06	3.04 ± 0.02	2.93 ± 0.13	2.76 ± 0.21	2.86 ± 0.06	2.94 ± 0.03
**Dorsal white muscle**						
Crude protein/%	10.01 ± 0.12^d^	13.01 ± 0.45^a^	13.36 ± 0.31^a^	12.49 ± 0.09^ab^	12.28 ± 0.34^b^	11.28 ± 0.51^c^
Crude lipid/%	2.33 ± 0.06^cd^	2.18 ± 0.06^d^	2.19 ± 0.02^d^	2.38 ± 0.05^c^	2.71 ± 0.53^b^	3.07 ± 0.06^a^
Moisture/%	77.37 ± 0.19	78.17 ± 0.40	78.59 ± 0.56	78.21 ± 0.24	77.46 ± 0.33	77.46 ± 0.33
Ash/%	1.24 ± 0.06	1.13 ± 0.04	1.16 ± 0.06	1.13 ± 0.03	1.14 ± 0.02	1.15 ± 0.06
**Hepatopancreas**						
Crude lipid/%	6.97 ± 0.32^d^	7.12 ± 0.19^d^	7.13 ± 0.16^d^	7.82 ± 0.04^c^	9.30 ± 0.19^b^	11.06 ± 0.19^a^
Moisture/%	72.45 ± 0.82	72.16 ± 0.44	74.65 ± 0.39	73.44 ± 0.65	70.69 ± 0.34	73.88 ± 0.25

* *Values are the mean ± SD (n = 3) of three replicates.*

*Values within the same row with different small letters in superscript are significantly different (p < 0.05)*.

### Serum Biochemical Parameters

Serum biochemical parameters showed that an obviously higher TCHO content was observed in the 180 g/kg dietary lipid treatment than in 30 and 60 g/kg dietary lipid treatments (*p* < 0.05). Fish fed the 120 g/kg crude lipid diet presented the lowest LDL-C content in serum, with a significant difference in 30 and 60 g/kg dietary lipid treatments (*p* < 0.05; Table 5).

**Table 5:** Serum biochemical parameters of large-sized common carp fed the diets containing different lipid levels^*^.

**Items**	**Varying levels of dietary lipid**					
	**30 g/kg**	**60 g/kg**	**90 g/kg**	**120 g/kg**	**150 g/kg**	**180 g/kg**
TG/(mM)^1^	0.96 ± 0.03	0.92 ± 0.06	0.81 ± 0.07	0.91 ± 0.07	0.96 ± 0.06	0.92 ± 0.09
TCHO/(mM)^2^	3.14 ± 0.21^b^	3.17 ± 0.15^b^	3.49 ± 0.09^ab^	3.37 ± 0.2^ab^	3.62 ± 0.25^ab^	3.85 ± 0.23^a^
HDL-C/(mM)^3^	1.64 ± 0.07	1.63 ± 0.06	1.74 ± 0.04	1.77 ± 0.07	1.67 ± 0.09	1.84 ± 0.09
LDL-C/(mM)^4^	0.80 ± 0.08^b^	0.85 ± 0.08^b^	0.97 ± 0.04^ab^	1.06 ± 0.11^ab^	1.12 ± 0.11^a^	0.94 ± 0.08^ab^

* *Values are the mean ± SD (n = 3) of three replicates.*

*Values within the same row with different small letters in superscript are significantly different (p < 0.05).*

*^1^Triacylglycerol (TG); ^2^Total cholesterol (TCHO); ^3^High-density lipoprotein-cholesterol (HDL-C); and ^4^Low-density lipoprotein-cholesterol (LDL-C)*.

### Relative Gene Expressions of the Growth Hormone/Insulin-Like Growth Factor-1 Axis

**Figure 3:**
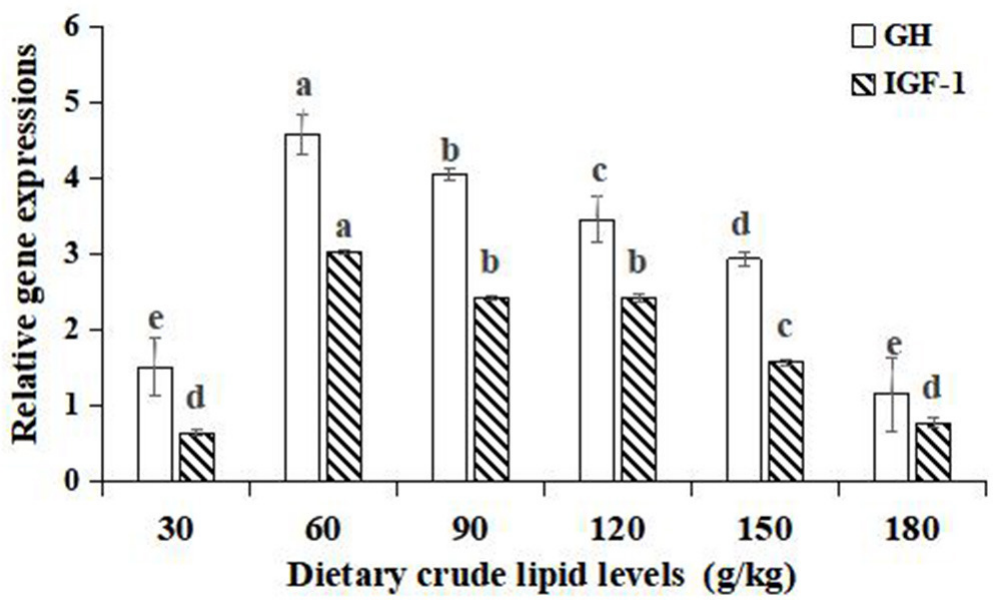
Relative gene expressions of the GH/IGF-1 axis in the hepatopancreas of large-sized common carp fed the diets containing different lipid levels. Lowercase letters (a, b, c, or d) indicate a significant effect of relative gene expressions of the GH/IGF-1 axis in the hepatopancreas (*p* < 0.05). GH, Growth hormone; IGF-1, Insulin growth factor-1.

Significant improvements in the relative gene expressions of growth hormone (GH) and insulin-like growth factor-1 (IGF-1) were found with an increase in the dietary lipid levels from 30 to 60 g/kg (*p* < 0.05), and further increases in the dietary lipid levels from 90 to 180 g/kg resulted in a significant decrease of relative gene expressions (*p* < 0.05). The peak values of relative gene expression of GH and IGF-1 were observed in the 60 g/kg dietary lipid treatment, with a significant difference in the other dietary lipid treatments (*p* < 0.05). The lowest GH and IGF-1 relative gene expression were both found in the 30 g/kg dietary lipid treatment, but no significant difference was observed compared to the 180 g/kg dietary lipid treatment (*p* > 0.05; Figure 3).

### Relative Gene Expressions of Lipid Metabolism

**Figure 4:**
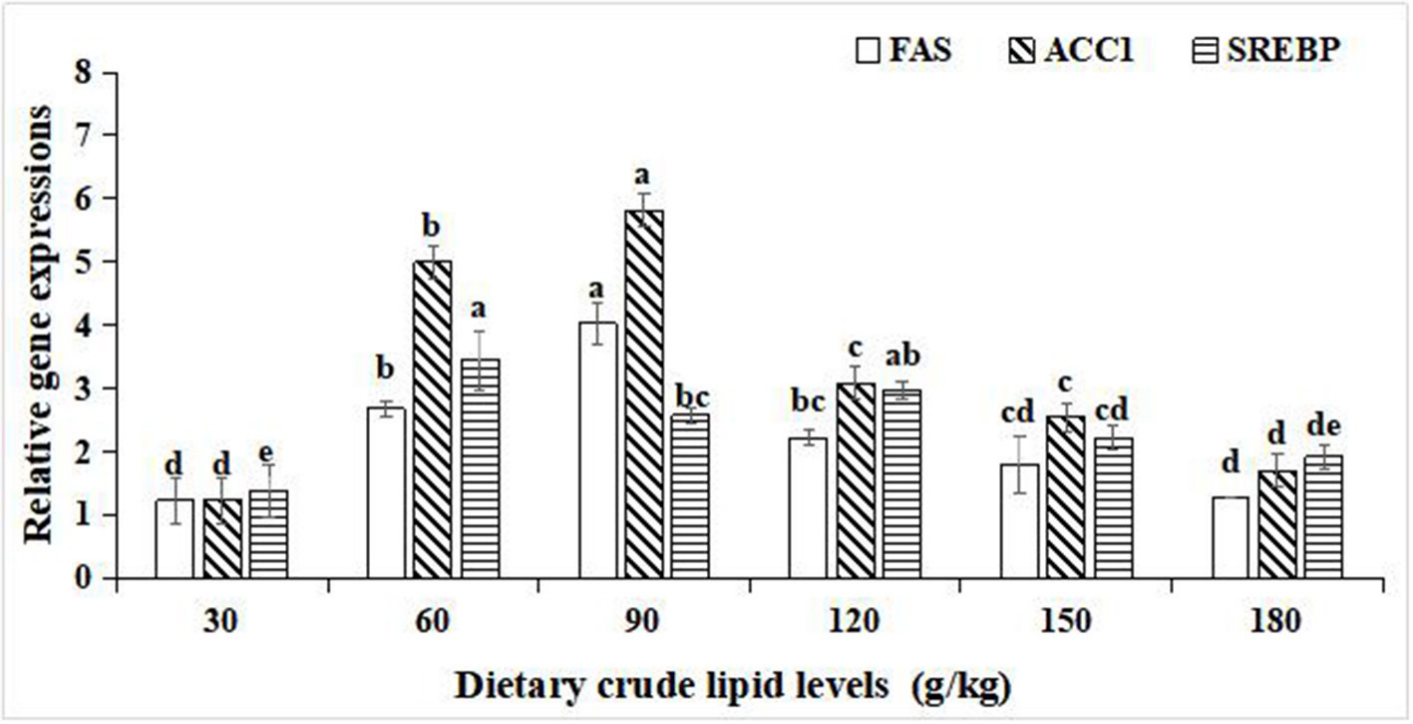
Relative gene expressions of lipid metabolism in the hepatopancreas of large-sized common carp fed the diets containing different lipid levels. Lowercase letters (a, b, c, or d) indicate a significant effect of relative gene expressions of lipid synthesis in the hepatopancreas (*p* < 0.05). FAS, Fatty acid synthase; SREBP, Sterol regulatory element binding protein; ACC-1, Acetyl-CoA carboxylase-1.

For the relative gene expression for lipid synthesis in the hepatopancreas, significant improvements in the relative gene expression of fatty acid synthase (FAS), acetyl-CoA carboxylase-1 (ACC-1), and sterol regulatory element binding protein (SREBP) were observed with an increase in dietary crude lipids from 30 to 90 g/kg (*p* < *0.05*), and a significant decrease in relative gene expression of FAS, ACC-1, and SREBP resulted in further increment of crude lipids to 120 and 180 g/kg (*p* < 0.05). Moreover, the peak values of the relative gene expression of FAS and ACC-1 were observed in the 90 g/kg dietary lipid treatment (*p* < 0.05). The peak value of relative gene expression of SREBP was observed in the 60 g/kg dietary lipid treatment, and it did not significantly differ from the 120 g/kg dietary lipid treatment (*p* < 0.05; Figure 4).

**Figure 5:**
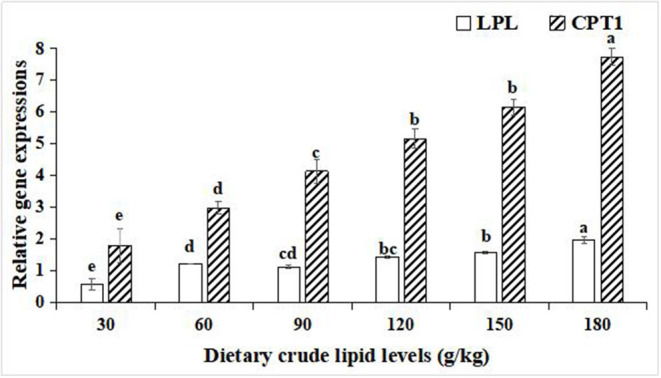
Relative gene expressions of lipid metabolism in the hepatopancreas of large-sized common carp fed the diets containing different lipid levels. Lowercase letters (a, b, c, or d) indicate a significant effect of relative gene expressions of lipid catabolism in the hepatopancreas (*p* < 0.05). LPL, Lipoprotein lipase; CPT1, Carnitine palmitoyltransferase-1.

For the relative gene expressions associated with lipid catabolism in the hepatopancreas, lipoprotein lipase (LPL), and carnitine palmitoyltransferase-1 (CPT1), relative gene expression levels in the hepatopancreas were both upregulated with an increase in the dietary lipid levels in the 180 g/kg dietary lipid treatment, and the peak values of the relative gene expression of LPL and CPT1 were observed in the 180 g/kg dietary lipid treatment, with a significant difference in the other dietary lipid treatments (*p* < 0.05; Figure 5).

## Discussion

A typical dose-response effect of dietary crude lipids on growth performance of large-sized common carp with an IBW of 247.00 ± 16.67 g was observed, with the highest WGR and SGR corresponding to fish fed the 60 g/kg lipid diets while a reduction in growth performance was found as the dietary crude lipid increased beyond this level. Analogously, a typical carnivorous marine fish *Argyrosomus regius* with an IBW of 229.7 ± 1.4 g fed a 170 g/kg lipid diet acquired the superior growth performance compared with fish fed 130 and 210 g/kg diets, and increasing the dietary lipid levels from 170 to 210 g/kg led to impaired growth performance (29). A typical herbivorous freshwater fish (*Ctenopharyngodon idella*) with an IBW of 261.41 ± 0.53 g showed a significantly higher SGR in fish fed a 36 g/kg lipid diet in comparison to those fed diets containing lipid levels of 5.9, 21.4, 66.6, or 80.1 g/kg (30). Based upon a series of discoveries, for the large-sized fish, we draw a conclusion that excessively low or high dietary lipid levels resulted in growth inhibition as a result of deficient or imbalanced nutrients. However, it is worth pointing out that the three fish species in the abovementioned studies belong to warm-water sciaenids. In sharp contrast to what has been described for warm-water sciaenids, there was an obvious linear relationship between the dietary lipid levels and growth performance in some cold-water species, such as large-sized triploid rainbow trout (*Oncorhynchus mykiss*) with an IBW of 233 g, which was reported to present a better growth performance when fed a 290 g/kg lipid diet rather than other lower dietary lipid levels (31), or Atlantic salmon (*Salmo salar*) with 1,026 g, which had higher growth rates when fed a 380–470 g/kg lipid diet, than when fed a 310 g/kg lipid diet (32). Consequently, when confirming the dietary lipid requirement, we should need to consider the biological characteristics of different fish species. From the standpoint of feed utilization, a deterioration in FCR was observed in fish fed high-lipid diets, which corresponded to the reduction of WGR and SGR. As it turned out, the FCR reached the maximum value (2.40 ± 0.03) in fish fed a 180 g/kg crude lipid diet, and was significantly higher compared to that of the fish in the other treatments. Simultaneously, a significant decline in PER was observed in fish fed a 180 g/kg crude lipid diet compared to fish in the other treatments. The above findings implied that the escalating dietary lipid levels exerted a passive influence on the nutrient utilization for fish, exactly as has been reported as the findings in the study on tench (*Tinca tinca* L.) with an IBW of 0.382 ± 0.08 g (33), common sole (*Solea solea*) with an IBW of 13.8 ± 0.4 g (12), common carp with an IBW of 36.12 ± 1.18 g (5), *A. regius* with an IBW of 229.7 ± 1.4 g (29). This discovery might also imply that a significant protein-sparing effect could not be effectually realized in many fish species by excessive dietary lipid supplements.

Based on the strength of the second-order polynomial regression analysis of WGR and FCR, the requirement of dietary lipids for large-sized common carp with an IBW of 247.00 ± 16.67 g was estimated to be 59.5–70.4 g/kg. This requirement is 10–30 g/kg lower than that observed in common carp with an IBW of 5.51 ± 0.05 g (24), common carp with an IBW of 15 g (26), and common carp with an IBW of 36.12 ± 1.18 g (5). The above difference supports somehow the hypothesis that there is a more excellent nutritional requirement in the juvenile stage. For one thing, small-sized fish is in a rapid growth period, so fish can use more dietary proteins for protein deposition to reduce the proportion of protein used for energy consumption. Hence, the diets of juvenile fish need more non-protein energy sources to meet the energy for body metabolism. However, large-sized fish is in a slow growth period, so appropriate diet lipid contents can promote the growth, whereas excessive dietary lipids will cause the liver steatosis and lipid oxidative stress, thereby deteriorating growth and health status of fish (27, 34). There exists, in addition, an obvious difference in lipid synthesis capacity between small-sized fish and large-sized fish. Phospholipid is a kind of lipid and an important component of lipoprotein. The lack of lipoprotein can lead to fat deposition in the liver. Therefore, the addition of 30–60 g/kg phospholipids in diets is necessary for the small-size fish. For large-sized fish, the ability to synthesize phospholipids will be strengthened, which implies that a large-sized fish has the stronger ability to synthesize lipid in comparison to a small-sized fish (35). The change trend of lipid metabolic regulation with fish weight needs to be lucubrated in future.

Apart from external manifestations, such as FCR, PER, and so on, internal manifestations, such as the protein relative gene expression of the GH/IGF axis in the muscle or liver and deposition in the whole body or muscle, play an irreplaceable role in hastening growth (36, 37). As expected in our study, in accordance with the change trend of growth performance, the hepatic GH and IGF-1 expression levels reached peaks with the dietary lipid levels up to 60 g/kg and did not increase further with higher lipid levels. A few studies on pejerrey (*Odontesthes bonariensis*) (38) and rainbow trout (31) have also revealed that the optimal dietary lipid level improved the growth of fish by promoting the proper functioning of the GH/IGF axis. Furthermore, the whole-body and muscle protein contents obtained the maximum levels with the dietary lipid levels up to 60 and 90 g/kg, respectively, and did not also increase further with higher lipid levels, indicating that dietary lipids would be important for the protein-sparing effect when ranging from 60 to 90 g/kg. These results nearly fell within the estimated optimal lipid level range of large-sized common carp (59.5–70.4 g/kg) based on the second-order polynomial regression analysis of WGR and FCR.

As already pointed out by Shearer (39), the lipid content of the whole body and tissues would be susceptible to some exogenous factors, especially diets. In the current study, the whole-body lipid content of the fish fed a 180 g/kg crude lipid diet reached the highest value although it was not significantly different from the other treatments. Furthermore, fish fed the 120–180 g/kg crude lipid diets obtained significantly higher muscle and hepatopancreas lipid contents than fish fed lower crude lipid treatments, and fish fed a 180 g/kg crude lipid diet obtained the highest muscle lipid content (30.7 ± 0.6 g/kg) and hepatopancreas lipid content (110.6 ± 1.9 g/kg). These findings obtained were similar to those obtained in other fish species, such as channel catfish (40), red drum (*Sciaenop socellata*) (41), rainbow trout (42), grass carp (43), and sea cucumber (*Apostichopus japonicus*) (44), verifying that undue dietary lipid supplements would cause excessive lipid deposition in the visceral cavity and tissues.

Because the crude lipid content in the muscle of large-sized common carp ranged from 23.3 to 30.7g/kg, in fish fed the 30 and 180 g/kg crude lipid diets, respectively, this species would be categorized as low-fat fish, according to the classification of fishes depending on their meat lipid content (45). This result provided a solid evidence for the fact that common carp has a restricted capacity for lipid deposition in the muscle, which was consistent with other species such as meager with an IBW of 229.7 ± 1.4 g (29). In contrast to meager with an IBW of 229.7 ± 1.4 g (29), the hepatopancreas was significantly affected by the dietary lipid levels since there were not only merely significant differences in the HSIs but also in the lipid content of hepatopancreas among the six treatments. The obtained result was similar to that obtained in a study on gilt-head sea bream (*Chrysophyrys auratus*), European seabass (*Dicentrarchus labrax*) (46), and *Chelon haematocheilus* (47), implying that the hepatopancreas (or say liver) was most likely to be a major lipid deposition site in common carp. Nevertheless, it should be pointed out that further research on the lipid deposition pattern among different fish species, fish sizes, and growth stages also needs to be strengthened.

Blood is the carrier of nutrients and metabolic waste in a live animal, and blood lipid can reflect the status of lipid metabolism *in vivo*. If a hepatocyte structure or the function of the liver is impaired, The concentrations of TG and TCHO in plasma will rise sharply (48). In the current study, despite no significant difference in TG contents among all treatments, significant improvements in the TCHO content of serum were observed with an increase in the dietary lipid levels, suggesting that an endogenous lipid transport remained in a state of animation. Different from our findings, research on *C. haematocheilus* reported a lack of significant differences in the concentrations of TG and TCHO in plasma (47). This discrepancy might be ascribed to a difference in the trend change of HDL-C contents. HDL-C is considered as a crucial tool for transporting the cholesterol from tissue cells back to liver for metabolic transformation (48). Research on *C. haematocheilus* found that HDL-C contents were significantly increased by an increase in the dietary lipid levels (47) while our research found that there was no significant difference among the six experimental groups in the HDL-C contents of serum. Hence, the shortage in HDL-C, which played a negative role in promptly removing excess cholesterol from blood and tissue cells, might be a major element in affecting the metabolic balance of blood lipid and liver lipid. As a method of alleviating the metabolic burden of liver lipid, the LDL-C in the blood should not be ignored because it is the primary vehicle for transporting cholesterol to extrahepatic tissues (48). In agreement to this, a significant increment in the LDL-C content of serum was registered in large-sized common carp with an increase in the dietary lipid levels. Combined with the result of molecular biology, these results seem to imply that the body is actively coping with the metabolic burden generated by high-lipid diets although the goal of reducing lipid deposition in the hepatopancreas has not been achieved. Hence, further research on improving lipid metabolism of the hepatopancreas in fish needs to be conducted.

In the hepatopancreas, the expression levels of lipogenic genes and lipolysis genes reflect the lipometabolic state for fish (49). In the present study, with an increase of the dietary lipid level from 30 to 90 g/kg, the expression levels of lipogenic genes FAS, ACC-1, and SREBP1 gradually increased, while with an increase in the dietary lipid levels from 120 to 180 g/kg, the expression levels of FAS, ACC-1, and SREBP1 significantly decreased. In addition, the expression levels of lipolysis genes LPL and CPT1 were lifted as the dietary lipid levels increased, but the expression levels of LPL and CPT1 in 30, 60,and 90 g/kg dietary lipid treatments were significantly lower than those of other three treatments. It was suggested that there were different metabolic adaptation mechanisms between high- and low-lipid diets for large-sized common carp. When fed the low-lipid diets, large-sized common carp would enhance the endogenous lipid synthesis of the hepatopancreas and relatively weaken the endogenous lipodysis to address an insufficient intake of exogenous lipid, and this was consistent with the results studied on grass carp fed low-lipid diets (50). In contrast, if faced with an excess of dietary lipid supplement, upregulation in the expression levels of FAS, ACC-1, and SREBP1 and relative downregulation in the expression levels of LPL and CPT1 in the hepatopancreas of large-sized common carp were conducted to maintain the dynamic and metabolic balance between the absorption and utilization of exogenous dietary lipids and endogenous lipid synthesis (51). Similar findings were reported in rainbow trout (52), GIFT tilapia (*Oreochromis niloticus*) (53), and *Onychostoma macrolepis* (54). In addition, it needs to be emphasized that the lowest expressions of lipogenic genes and lipolysis genes in the hepatopancreas were observed in the 30 g/kg treatment, indicating that a restrictive and an insufficient lipid intake induced to nutritional dysregulation stress for large-sized common carp (55).

## Conclusion

Excessive amount of dietary crude lipid generates a lipid metabolic burden for large-sized common carp and results in worsening of growth performance and feed utilization efficiency, the higher TCHO contents in serum, the higher muscle and hepatopancreas lipid deposition, and a less-than-ideal protein-sparing effect. The results revealed that an excessive amount of dietary lipid supplements (>60 mg/kg) would inhibit WG and aggravate the lipid decomposition in the hepatopancreas. Based on WGR and FCR, the dietary lipid levels of 59.5 and 70.4 g/kg were optimal for growth performance and feed utilization of large-sized common carp. This should be taken into consideration in formulating specific practical diets for large-sized common carp.

## Data Availability Statement

The original contributions presented in the study are included in the article/supplementary material, further inquiries can be directed to the corresponding author/s.

## Ethics Statement

The animal study was reviewed and approved by the Committee for the Welfare and Ethics of Laboratory Animals of Heilongjiang River Fisheries Research Institute, CAFS.

## Author Contributions

LW: Conceptualization, investigation, writing–review and editing. ZF: Writing the original draft preparation, formal analysis and resources. DW: Formal analysis and investigation. JL, YZ, and CW: Formal analysis. XZ: Project administration and investigation. All authors contributed to the article and approved the submitted version.

## Conflict of Interest

The authors declare that the research was conducted in the absence of any commercial or financial relationships that could be construed as a potential conflict of interest.
